# 5-Cyclo­pentyl-3-methyl­sulfinyl-2-phenyl-1-benzofuran

**DOI:** 10.1107/S1600536811010920

**Published:** 2011-03-31

**Authors:** Hong Dae Choi, Pil Ja Seo, Byeng Wha Son, Uk Lee

**Affiliations:** a Department of Chemistry, Dongeui University, San 24 Kaya-dong Busanjin-gu, Busan 614-714, Republic of Korea; b Department of Chemistry, Pukyong National University, 599-1 Daeyeon 3-dong, Nam-gu, Busan 608-737, Republic of Korea

## Abstract

In the title compound, C_20_H_20_O_2_S, the cyclo­pentyl ring adopts an envelope conformation with the flap atom connected to the benzofuran residue. The phenyl ring makes a dihedral angle of 32.36 (9)° with the mean plane of the benzofuran fragment. In the crystal, mol­ecules are linked through weak C—H⋯O inter­actions. In the cyclo­pentyl ring, two adjacent C atoms are disordered over two sets of sites with site occupancy factors of 0.675 (8) and 0.325 (8).

## Related literature

For the biological activity of benzofuran compounds, see: Aslam *et al.* (2006 ▶); Galal *et al.* (2009 ▶); Khan *et al.* (2005 ▶). For natural products with benzofuran rings, see: Akgul & Anil (2003 ▶); Soekamto *et al.* (2003 ▶). For structural studies of related 2-aryl-5-cyclo­hexyl-3-methyl­sulfinyl-1-benzofuran derivatives, see: Choi *et al.* (2011**a* ▶,b* ▶).
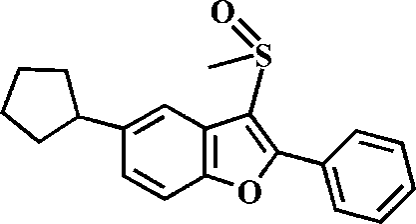


## Experimental

### 

#### Crystal data

C_20_H_20_O_2_S
*M* *_r_* = 324.42
Monoclinic, 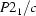

*a* = 5.9586 (7) Å
*b* = 25.397 (3) Å
*c* = 10.7804 (12) Å
β = 90.038 (2)°
*V* = 1631.4 (3) Å^3^
*Z* = 4
Mo *K*α radiation
μ = 0.21 mm^−1^
*T* = 173 K
0.34 × 0.15 × 0.12 mm

#### Data collection

Bruker SMART APEXII CCD diffractometer
Absorption correction: multi-scan (*SADABS*; Bruker, 2009 ▶) *T*_min_ = 0.934, *T*_max_ = 0.976
15888 measured reflections
3749 independent reflections
2998 reflections with *I* > 2σ(*I*)
*R*_int_ = 0.043

#### Refinement

*R*[*F* ^2^ > 2σ(*F* ^2^)] = 0.055
*wR*(*F* ^2^) = 0.139
*S* = 1.06
3749 reflections
228 parameters
36 restraints
H-atom parameters constrained
Δρ_max_ = 0.42 e Å^−3^
Δρ_min_ = −0.37 e Å^−3^

### 

Data collection: *APEX2* (Bruker, 2009 ▶); cell refinement: *SAINT* (Bruker, 2009 ▶); data reduction: *SAINT*; program(s) used to solve structure: *SHELXS97* (Sheldrick, 2008 ▶); program(s) used to refine structure: *SHELXL97* (Sheldrick, 2008 ▶); molecular graphics: *ORTEP-3* (Farrugia, 1997 ▶) and *DIAMOND* (Brandenburg, 1998 ▶); software used to prepare material for publication: *SHELXL97*.

## Supplementary Material

Crystal structure: contains datablocks global, I. DOI: 10.1107/S1600536811010920/tk2730sup1.cif

Structure factors: contains datablocks I. DOI: 10.1107/S1600536811010920/tk2730Isup2.hkl

Additional supplementary materials:  crystallographic information; 3D view; checkCIF report

## Figures and Tables

**Table 1:** Hydrogen-bond geometry (Å, °)

*D*—H⋯*A*	*D*—H	H⋯*A*	*D*⋯*A*	*D*—H⋯*A*
C19—H19⋯O2^i^	0.95	2.53	3.324 (3)	142

Symmetry code: (i) 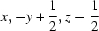.

**Fig. 1.**
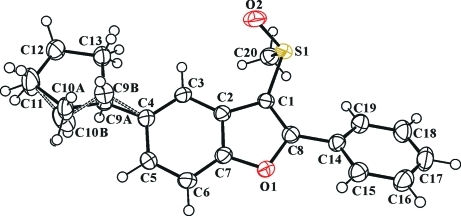
The molecular structure of the title compound with the atom numbering scheme. Displacement ellipsoids are drawn at the 50% probability level. H atoms are presented as a small spheres of arbitrary radius.

## supplementary crystallographic information

### Comment

Many compounds having a benzofuran skeleton have attracted much attention due to their pharmacological properties such as antifungal, antimicrobial, antitumor and antiviral activities (Aslam *et al.*, 2006; Galal *et al.*, 2009; Khan *et al.*, 2005). These compounds occur in a wide range of natural products (Akgul & Anil, 2003; Soekamto *et al.*, 2003). As part of our ongoing program investigating the substituent effect on the solid state structures of 2-aryl-5-cyclohexyl-3-methylsulfinyl-1-benzofuran analogues (Choi *et al.*, 2011**a*, b*), we report herein on the crystal structure of the title compound.

In the title molecule (Fig. 1), the benzofuran unit is essentially planar, with a mean deviation of 0.012 (2) Å from the least-squares plane defined by the nine constituent atoms. The cyclopentyl ring is in the envelope form. In the cyclopentyl ring, two C atoms (C9 & C10) are disordered over two positions with site-occupancy factors, from refinement of 0.675 (8) (part A) and 0.325 (8) (part B). The phenyl ring makes a dihedral angle of 32.36 (9)° with the mean plane of the benzofuran ring. The crystal packing is stabilized by a weak intermolecular C—H···O hydrogen bond between a phenyl-H atom and the oxygen of the S═O unit (Table 1; C19—H19···O2^i^).

### Experimental

77% 3-Chloroperoxybenzoic acid (269 mg, 1.2 mmol) was added in small portions to a stirred solution of 5-cyclopentyl-3-methylsulfanyl-2-phenyl-1-benzofuran (339 mg, 1.1 mmol) in dichloromethane (30 mL) at 273 K. After being stirred at room temperature for 3 h, the mixture was washed with saturated sodium bicarbonate solution and the organic layer was separated, dried over magnesium sulfate, filtered and concentrated at reduced pressure. The residue was purified by column chromatography (hexane–ethyl acetate, 2:1 v/v) to afford the title compound as a colorless solid [yield 73%, *M*.pt. 415–416 K; *R*_f_ = 0.61 (hexane–ethyl acetate, 2:1 v/v)]. Single crystals suitable for X-ray diffraction were prepared by slow evaporation of an acetone solution of the title compound held at room temperature.

### Refinement

All H atoms were positioned geometrically and refined using a riding model, with C—H = 0.95 Å for aryl, 1.00 Å for methine, 0.99 Å for methylene and 0.98 Å for methyl H atoms, respectively. *U*_iso_(H) =1.2*U*_eq_(C) for aryl-, methine- and methylene-H, and 1.5*U*_eq_(C) for methyl-H atoms. Two C atoms of the cyclopentyl ring are disordered over two positions with site occupancy factors, from refinement, of 0.675 (8) (part A) and 0.325 (8) (part B). The distance of equivalent C—C pairs was restrained to 0.001 Å using command DFIX and SADI, and displacement ellipsoids of C9 and C10 set were restrained to 0.01 using commend ISOR.

### Crystal data

**Table:**

C_20_H_20_O_2_S	*F*(000) = 688
*M**_r_* = 324.42	*D*_x_ = 1.321 Mg m^−^^3^
Monoclinic, *P*2_1_/*c*	Mo *K*α radiation, λ = 0.71073 Å
Hall symbol: -P 2ybc	Cell parameters from 3689 reflections
*a* = 5.9586 (7) Å	θ = 2.5–26.8°
*b* = 25.397 (3) Å	µ = 0.21 mm^−^^1^
*c* = 10.7804 (12) Å	*T* = 173 K
β = 90.038 (2)°	Block, colourless
*V* = 1631.4 (3) Å^3^	0.34 × 0.15 × 0.12 mm
*Z* = 4	

### Data collection

**Table:**

Bruker SMART APEXII CCD diffractometer	3749 independent reflections
Radiation source: rotating anode	2998 reflections with *I* > 2σ(*I*)
graphite multilayer	*R*_int_ = 0.043
Detector resolution: 10.0 pixels mm^-1^	θ_max_ = 27.6°, θ_min_ = 1.6°
φ and ω scans	*h* = −7→7
Absorption correction: multi-scan (*SADABS*; Bruker, 2009)	*k* = −33→32
*T*_min_ = 0.934, *T*_max_ = 0.976	*l* = −14→13
15888 measured reflections	

### Refinement

**Table:**

Refinement on *F*^2^	Primary atom site location: structure-invariant direct methods
Least-squares matrix: full	Secondary atom site location: difference Fourier map
*R*[*F*^2^ > 2σ(*F*^2^)] = 0.055	Hydrogen site location: difference Fourier map
*wR*(*F*^2^) = 0.139	H-atom parameters constrained
*S* = 1.06	*w* = 1/[σ^2^(*F*_o_^2^) + (0.0448*P*)^2^ + 1.8445*P*] where *P* = (*F*_o_^2^ + 2*F*_c_^2^)/3
3749 reflections	(Δ/σ)_max_ < 0.001
228 parameters	Δρ_max_ = 0.42 e Å^−^^3^
36 restraints	Δρ_min_ = −0.37 e Å^−^^3^

### Special details

**Table:**

Geometry. All esds (except the esd in the dihedral angle between two l.s. planes) are estimated using the full covariance matrix. The cell esds are taken into account individually in the estimation of esds in distances, angles and torsion angles; correlations between esds in cell parameters are only used when they are defined by crystal symmetry. An approximate (isotropic) treatment of cell esds is used for estimating esds involving l.s. planes.
Refinement. Refinement of F^2^ against ALL reflections. The weighted R-factor wR and goodness of fit S are based on F^2^, conventional R-factors R are based on F, with F set to zero for negative F^2^. The threshold expression of F^2^ > 2sigma(F^2^) is used only for calculating R-factors(gt) etc. and is not relevant to the choice of reflections for refinement. R-factors based on F^2^ are statistically about twice as large as those based on F, and R- factors based on ALL data will be even larger.

### Fractional atomic coordinates and isotropic or equivalent isotropic displacement parameters (Å^2^)

**Table:**

	*x*	*y*	*z*	*U*_iso_*/*U*_eq_	Occ. (<1)
S1	−0.00822 (10)	0.27762 (2)	0.59148 (5)	0.03089 (17)	
O1	−0.0724 (3)	0.43153 (6)	0.54750 (15)	0.0316 (4)	
O2	0.0353 (4)	0.26204 (7)	0.72244 (17)	0.0452 (5)	
C1	0.0137 (4)	0.34662 (9)	0.5858 (2)	0.0274 (5)	
C2	0.1643 (4)	0.37906 (9)	0.6581 (2)	0.0291 (5)	
C3	0.3432 (4)	0.37030 (9)	0.7400 (2)	0.0348 (5)	
H3	0.3856	0.3354	0.7616	0.042*	
C4	0.4575 (5)	0.41296 (10)	0.7891 (3)	0.0429 (6)	
C5	0.3859 (5)	0.46426 (10)	0.7584 (2)	0.0429 (6)	
H5	0.4628	0.4934	0.7939	0.051*	
C6	0.2092 (4)	0.47403 (9)	0.6792 (2)	0.0364 (6)	
H6	0.1631	0.5088	0.6592	0.044*	
C7	0.1034 (4)	0.43049 (9)	0.6309 (2)	0.0300 (5)	
C8	−0.1229 (4)	0.37977 (9)	0.5210 (2)	0.0294 (5)	
C9A	0.6772 (5)	0.40623 (12)	0.8627 (3)	0.0267 (9)	0.675 (8)
H9A	0.8081	0.4161	0.8096	0.032*	0.675 (8)
C9B	0.5876 (14)	0.4037 (2)	0.9106 (5)	0.052 (3)	0.325 (8)
H9B	0.4761	0.4041	0.9799	0.063*	0.325 (8)
C10A	0.6756 (8)	0.44015 (19)	0.9815 (4)	0.0447 (13)	0.675 (8)
H10A	0.5346	0.4354	1.0287	0.054*	0.675 (8)
H10B	0.6956	0.4779	0.9620	0.054*	0.675 (8)
C10B	0.7683 (15)	0.4454 (4)	0.9408 (8)	0.054 (3)	0.325 (8)
H10C	0.7012	0.4797	0.9635	0.065*	0.325 (8)
H10D	0.8749	0.4502	0.8713	0.065*	0.325 (8)
C11	0.8799 (6)	0.41795 (13)	1.0536 (4)	0.0754 (12)	
H11A	1.0166	0.4387	1.0354	0.091*	0.675 (8)
H11B	0.8519	0.4189	1.1441	0.091*	0.675 (8)
H11C	1.0272	0.4340	1.0732	0.091*	0.325 (8)
H11D	0.7826	0.4198	1.1279	0.091*	0.325 (8)
C12	0.9068 (5)	0.36158 (11)	1.0090 (3)	0.0431 (6)	
H12A	0.8976	0.3367	1.0796	0.052*	
H12B	1.0534	0.3568	0.9673	0.052*	
C13	0.7140 (5)	0.35212 (10)	0.9178 (3)	0.0417 (6)	
H13A	0.7567	0.3263	0.8532	0.050*	0.675 (8)
H13B	0.5779	0.3394	0.9612	0.050*	0.675 (8)
H13C	0.7728	0.3422	0.8352	0.050*	0.325 (8)
H13D	0.6146	0.3236	0.9481	0.050*	0.325 (8)
C14	−0.3000 (4)	0.37178 (9)	0.4292 (2)	0.0305 (5)	
C15	−0.4765 (4)	0.40780 (10)	0.4209 (2)	0.0356 (5)	
H15	−0.4818	0.4372	0.4752	0.043*	
C16	−0.6434 (4)	0.40057 (12)	0.3334 (3)	0.0444 (6)	
H16	−0.7626	0.4253	0.3272	0.053*	
C17	−0.6375 (5)	0.35756 (13)	0.2550 (3)	0.0483 (7)	
H17	−0.7534	0.3524	0.1957	0.058*	
C18	−0.4623 (5)	0.32203 (12)	0.2631 (2)	0.0448 (6)	
H18	−0.4584	0.2925	0.2091	0.054*	
C19	−0.2934 (4)	0.32909 (10)	0.3487 (2)	0.0366 (5)	
H19	−0.1724	0.3048	0.3527	0.044*	
C20	0.2414 (4)	0.26247 (10)	0.5071 (2)	0.0387 (6)	
H20A	0.3681	0.2819	0.5428	0.058*	
H20B	0.2221	0.2727	0.4201	0.058*	
H20C	0.2710	0.2246	0.5121	0.058*	

### Atomic displacement parameters (Å^2^)

**Table:**

	*U*^11^	*U*^22^	*U*^33^	*U*^12^	*U*^13^	*U*^23^
S1	0.0364 (3)	0.0241 (3)	0.0322 (3)	−0.0045 (2)	−0.0029 (2)	0.0024 (2)
O1	0.0363 (9)	0.0261 (8)	0.0324 (9)	0.0025 (7)	−0.0072 (7)	0.0017 (6)
O2	0.0682 (13)	0.0342 (10)	0.0330 (10)	−0.0025 (9)	0.0011 (9)	0.0087 (8)
C1	0.0324 (12)	0.0235 (10)	0.0262 (11)	−0.0018 (8)	−0.0006 (9)	0.0013 (8)
C2	0.0358 (12)	0.0264 (11)	0.0251 (11)	−0.0014 (9)	−0.0013 (9)	0.0008 (9)
C3	0.0471 (14)	0.0244 (11)	0.0328 (12)	0.0012 (10)	−0.0112 (11)	0.0026 (9)
C4	0.0603 (17)	0.0283 (12)	0.0400 (14)	−0.0025 (11)	−0.0204 (13)	0.0010 (10)
C5	0.0616 (17)	0.0264 (12)	0.0406 (14)	−0.0038 (11)	−0.0191 (13)	−0.0029 (10)
C6	0.0496 (15)	0.0241 (11)	0.0354 (13)	0.0012 (10)	−0.0070 (11)	0.0015 (10)
C7	0.0363 (12)	0.0284 (11)	0.0254 (11)	0.0013 (9)	−0.0025 (9)	0.0008 (9)
C8	0.0314 (11)	0.0295 (12)	0.0273 (11)	−0.0011 (9)	0.0011 (9)	0.0006 (9)
C9A	0.0252 (17)	0.0292 (18)	0.0258 (18)	−0.0023 (13)	0.0028 (13)	0.0030 (13)
C9B	0.062 (6)	0.046 (5)	0.050 (5)	−0.011 (4)	−0.016 (5)	0.003 (4)
C10A	0.041 (3)	0.034 (2)	0.059 (3)	0.010 (2)	−0.026 (2)	−0.014 (2)
C10B	0.030 (5)	0.045 (5)	0.087 (7)	0.002 (4)	−0.002 (5)	−0.018 (5)
C11	0.078 (2)	0.0490 (19)	0.100 (3)	0.0152 (17)	−0.061 (2)	−0.0193 (18)
C12	0.0372 (14)	0.0417 (15)	0.0505 (16)	0.0036 (11)	−0.0111 (12)	0.0004 (12)
C13	0.0458 (15)	0.0351 (13)	0.0442 (15)	0.0034 (11)	−0.0128 (12)	−0.0053 (11)
C14	0.0296 (11)	0.0348 (12)	0.0271 (11)	−0.0022 (9)	−0.0014 (9)	0.0043 (9)
C15	0.0317 (12)	0.0386 (13)	0.0366 (13)	0.0008 (10)	0.0009 (10)	0.0031 (10)
C16	0.0315 (13)	0.0531 (17)	0.0485 (16)	0.0027 (12)	−0.0036 (11)	0.0111 (13)
C17	0.0366 (14)	0.067 (2)	0.0408 (15)	−0.0094 (13)	−0.0133 (12)	0.0061 (14)
C18	0.0473 (16)	0.0527 (17)	0.0343 (14)	−0.0065 (13)	−0.0079 (12)	−0.0051 (12)
C19	0.0366 (13)	0.0394 (14)	0.0338 (13)	−0.0003 (10)	−0.0040 (10)	−0.0007 (10)
C20	0.0446 (14)	0.0322 (13)	0.0395 (14)	0.0034 (11)	−0.0004 (11)	−0.0018 (10)

### Geometric parameters (Å, °)

**Table:**

S1—O2	1.4888 (19)	C10B—H10C	0.9900
S1—C1	1.758 (2)	C10B—H10D	0.9900
S1—C20	1.786 (3)	C11—C12	1.519 (4)
O1—C8	1.379 (3)	C11—H11A	0.9900
O1—C7	1.380 (3)	C11—H11B	0.9900
C1—C8	1.363 (3)	C11—H11C	0.9900
C1—C2	1.446 (3)	C11—H11D	0.9900
C2—C7	1.387 (3)	C12—C13	1.530 (4)
C2—C3	1.402 (3)	C12—H12A	0.9900
C3—C4	1.385 (3)	C12—H12B	0.9900
C3—H3	0.9500	C13—H13A	0.9900
C4—C5	1.410 (4)	C13—H13B	0.9900
C4—C9B	1.539 (4)	C13—H13C	0.9900
C4—C9A	1.540 (4)	C13—H13D	0.9900
C5—C6	1.377 (4)	C14—C19	1.389 (3)
C5—H5	0.9500	C14—C15	1.397 (3)
C6—C7	1.375 (3)	C15—C16	1.382 (4)
C6—H6	0.9500	C15—H15	0.9500
C8—C14	1.461 (3)	C16—C17	1.382 (4)
C9A—C13	1.513 (4)	C16—H16	0.9500
C9A—C10A	1.544 (4)	C17—C18	1.383 (4)
C9A—H9A	1.0000	C17—H17	0.9500
C9B—C13	1.513 (4)	C18—C19	1.377 (4)
C9B—C10B	1.543 (4)	C18—H18	0.9500
C9B—H9B	1.0000	C19—H19	0.9500
C10A—C11	1.550 (2)	C20—H20A	0.9800
C10A—H10A	0.9900	C20—H20B	0.9800
C10A—H10B	0.9900	C20—H20C	0.9800
C10B—C11	1.551 (2)		
			
O2—S1—C1	106.54 (11)	H11A—C11—H11B	108.8
O2—S1—C20	106.33 (12)	C12—C11—H11C	111.2
C1—S1—C20	97.78 (11)	C10A—C11—H11C	130.7
C8—O1—C7	106.39 (17)	C10B—C11—H11C	111.2
C8—C1—C2	107.1 (2)	H11A—C11—H11C	25.0
C8—C1—S1	126.08 (18)	H11B—C11—H11C	86.0
C2—C1—S1	126.56 (17)	C12—C11—H11D	111.2
C7—C2—C3	118.8 (2)	C10A—C11—H11D	85.9
C7—C2—C1	105.1 (2)	C10B—C11—H11D	111.2
C3—C2—C1	136.1 (2)	H11A—C11—H11D	128.2
C4—C3—C2	119.4 (2)	H11B—C11—H11D	26.2
C4—C3—H3	120.3	H11C—C11—H11D	109.1
C2—C3—H3	120.3	C11—C12—C13	105.8 (2)
C3—C4—C5	119.0 (2)	C11—C12—H12A	110.6
C3—C4—C9B	117.0 (3)	C13—C12—H12A	110.6
C5—C4—C9B	119.5 (3)	C11—C12—H12B	110.6
C3—C4—C9A	121.9 (2)	C13—C12—H12B	110.6
C5—C4—C9A	118.7 (2)	H12A—C12—H12B	108.7
C9B—C4—C9A	28.0 (3)	C9A—C13—C9B	28.5 (3)
C6—C5—C4	122.9 (2)	C9A—C13—C12	102.6 (2)
C6—C5—H5	118.6	C9B—C13—C12	105.7 (3)
C4—C5—H5	118.6	C9A—C13—H13A	111.2
C7—C6—C5	116.1 (2)	C9B—C13—H13A	131.7
C7—C6—H6	122.0	C12—C13—H13A	111.2
C5—C6—H6	122.0	C9A—C13—H13B	111.2
C6—C7—O1	125.4 (2)	C9B—C13—H13B	84.2
C6—C7—C2	123.9 (2)	C12—C13—H13B	111.2
O1—C7—C2	110.7 (2)	H13A—C13—H13B	109.2
C1—C8—O1	110.6 (2)	C9A—C13—H13C	86.0
C1—C8—C14	133.8 (2)	C9B—C13—H13C	110.6
O1—C8—C14	115.5 (2)	C12—C13—H13C	110.6
C13—C9A—C4	115.2 (2)	H13A—C13—H13C	26.7
C13—C9A—C10A	100.5 (3)	H13B—C13—H13C	129.2
C4—C9A—C10A	111.1 (2)	C9A—C13—H13D	134.9
C13—C9A—H9A	109.9	C9B—C13—H13D	110.6
C4—C9A—H9A	109.9	C12—C13—H13D	110.6
C10A—C9A—H9A	109.9	H13A—C13—H13D	84.4
C13—C9B—C4	115.2 (2)	H13B—C13—H13D	28.0
C13—C9B—C10B	103.6 (6)	H13C—C13—H13D	108.7
C4—C9B—C10B	115.2 (5)	C19—C14—C15	119.5 (2)
C13—C9B—H9B	107.5	C19—C14—C8	120.7 (2)
C4—C9B—H9B	107.5	C15—C14—C8	119.8 (2)
C10B—C9B—H9B	107.5	C16—C15—C14	119.9 (2)
C9A—C10A—C11	102.0 (3)	C16—C15—H15	120.1
C9A—C10A—H10A	111.4	C14—C15—H15	120.1
C11—C10A—H10A	111.4	C17—C16—C15	120.3 (3)
C9A—C10A—H10B	111.4	C17—C16—H16	119.9
C11—C10A—H10B	111.4	C15—C16—H16	119.9
H10A—C10A—H10B	109.2	C16—C17—C18	119.8 (3)
C9B—C10B—C11	99.0 (3)	C16—C17—H17	120.1
C9B—C10B—H10C	112.0	C18—C17—H17	120.1
C11—C10B—H10C	112.0	C19—C18—C17	120.6 (3)
C9B—C10B—H10D	112.0	C19—C18—H18	119.7
C11—C10B—H10D	112.0	C17—C18—H18	119.7
H10C—C10B—H10D	109.6	C18—C19—C14	120.0 (2)
C12—C11—C10A	105.5 (3)	C18—C19—H19	120.0
C12—C11—C10B	102.7 (5)	C14—C19—H19	120.0
C10A—C11—C10B	26.8 (3)	S1—C20—H20A	109.5
C12—C11—H11A	110.6	S1—C20—H20B	109.5
C10A—C11—H11A	110.6	H20A—C20—H20B	109.5
C10B—C11—H11A	87.6	S1—C20—H20C	109.5
C12—C11—H11B	110.6	H20A—C20—H20C	109.5
C10A—C11—H11B	110.6	H20B—C20—H20C	109.5
C10B—C11—H11B	133.6		
			
O2—S1—C1—C8	−139.2 (2)	C5—C4—C9B—C13	−162.9 (5)
C20—S1—C1—C8	111.2 (2)	C9A—C4—C9B—C13	−66.5 (3)
O2—S1—C1—C2	34.2 (2)	C3—C4—C9B—C10B	161.8 (6)
C20—S1—C1—C2	−75.4 (2)	C5—C4—C9B—C10B	−42.3 (8)
C8—C1—C2—C7	0.5 (2)	C9A—C4—C9B—C10B	54.1 (9)
S1—C1—C2—C7	−173.96 (17)	C13—C9A—C10A—C11	45.1 (4)
C8—C1—C2—C3	−178.4 (3)	C4—C9A—C10A—C11	167.5 (3)
S1—C1—C2—C3	7.2 (4)	C13—C9B—C10B—C11	−45.0 (8)
C7—C2—C3—C4	−1.5 (4)	C4—C9B—C10B—C11	−171.7 (5)
C1—C2—C3—C4	177.2 (3)	C9A—C10A—C11—C12	−26.3 (5)
C2—C3—C4—C5	2.1 (4)	C9A—C10A—C11—C10B	61.3 (9)
C2—C3—C4—C9B	158.0 (4)	C9B—C10B—C11—C12	45.7 (7)
C2—C3—C4—C9A	−170.2 (2)	C9B—C10B—C11—C10A	−53.6 (5)
C3—C4—C5—C6	−1.4 (5)	C10A—C11—C12—C13	−2.3 (4)
C9B—C4—C5—C6	−156.7 (5)	C10B—C11—C12—C13	−29.8 (5)
C9A—C4—C5—C6	171.1 (3)	C4—C9A—C13—C9B	−66.7 (3)
C4—C5—C6—C7	0.2 (4)	C10A—C9A—C13—C9B	52.8 (5)
C5—C6—C7—O1	−178.1 (2)	C4—C9A—C13—C12	−166.4 (3)
C5—C6—C7—C2	0.3 (4)	C10A—C9A—C13—C12	−47.0 (3)
C8—O1—C7—C6	178.3 (2)	C4—C9B—C13—C9A	66.7 (3)
C8—O1—C7—C2	−0.3 (2)	C10B—C9B—C13—C9A	−60.1 (7)
C3—C2—C7—C6	0.3 (4)	C4—C9B—C13—C12	154.3 (5)
C1—C2—C7—C6	−178.8 (2)	C10B—C9B—C13—C12	27.5 (6)
C3—C2—C7—O1	179.0 (2)	C11—C12—C13—C9A	30.8 (3)
C1—C2—C7—O1	−0.1 (3)	C11—C12—C13—C9B	1.6 (5)
C2—C1—C8—O1	−0.7 (3)	C1—C8—C14—C19	−30.9 (4)
S1—C1—C8—O1	173.79 (16)	O1—C8—C14—C19	146.6 (2)
C2—C1—C8—C14	176.8 (2)	C1—C8—C14—C15	150.1 (3)
S1—C1—C8—C14	−8.7 (4)	O1—C8—C14—C15	−32.5 (3)
C7—O1—C8—C1	0.6 (2)	C19—C14—C15—C16	0.4 (4)
C7—O1—C8—C14	−177.40 (19)	C8—C14—C15—C16	179.5 (2)
C3—C4—C9A—C13	−21.6 (4)	C14—C15—C16—C17	0.6 (4)
C5—C4—C9A—C13	166.1 (3)	C15—C16—C17—C18	−0.8 (4)
C9B—C4—C9A—C13	66.6 (3)	C16—C17—C18—C19	0.0 (4)
C3—C4—C9A—C10A	−135.0 (4)	C17—C18—C19—C14	1.0 (4)
C5—C4—C9A—C10A	52.7 (4)	C15—C14—C19—C18	−1.2 (4)
C9B—C4—C9A—C10A	−46.8 (5)	C8—C14—C19—C18	179.7 (2)
C3—C4—C9B—C13	41.3 (8)		

### Hydrogen-bond geometry (Å, °)

**Table:**

*D*—H···*A*	*D*—H	H···*A*	*D*···*A*	*D*—H···*A*
C19—H19···O2^i^	0.95	2.53	3.324 (3)	142

Symmetry codes: (i) *x*, −*y*+1/2, *z*−1/2.
